# Performance of gradient diffusion strip and disk diffusion versus broth microdilution for lefamulin susceptibility testing of 422 *Staphylococcus aureus* isolates

**DOI:** 10.1128/jcm.01578-25

**Published:** 2026-01-23

**Authors:** Yujing Tian, Xiangning He, Xue Wu, Yanjun Liu, Xinying Wang, Yan Jin, Guojun Wang, Zhijun Zhang

**Affiliations:** 1 Department of Laboratory Medicine, The Affiliated Taian City Central Hospital of Qingdao University, Taian, China; 2 Shandong Provincial Key Medical and Health Laboratory of Anti-Drug Resistant Drug Research, The Affiliated Taian City Central Hospital of Qingdao University, Taian, China; 3 Department of Neurosurgery, The Affiliated Taian City Central Hospital of Qingdao University, Taian, China; 4 Department of Brain Injury Laboratory, The Affiliated Taian City Central Hospital of Qingdao University, Taian, China; 5 Department of Neuroimmunology and Infectious Diseases, The Affiliated Taian City Central Hospital of Qingdao University, Taian, China; 6 Department of Pain Management, The Affiliated Taian City Central Hospital of Qingdao University, Taian, China; 7 Department of Laboratory Medicine, Shandong Provincial Hospital Affiliated to Shandong First Medical University, Shandong Provincial Hospital 518873, Jinan, China; Children's Hospital Los Angeles, Los Angeles, California, USA

**Keywords:** *Staphylococcus aureus*, lefamulin, antimicrobial susceptibility testing, broth microdilution, gradient diffusion strip, disk diffusion

## Abstract

Lefamulin is a novel pleuromutilin antibiotic with potent activity against methicillin-resistant *Staphylococcus aureus* (MRSA). While broth microdilution (BMD) is the reference method for susceptibility testing, simpler alternatives such as MIC Test Strip (gradient diffusion strip [GDS]) and disk diffusion (DD) are urgently needed for routine laboratories. We evaluated the accuracy of GDS and DD compared to BMD for lefamulin against *S. aureus*. A total of 422 non-duplicate clinical isolates of *S. aureus* (256 methicillin-susceptible *S. aureus* [MSSA] and 166 MRSA) collected between December 2023 and December 2024 at the Affiliated Tai’an Central Hospital of Qingdao University were tested by BMD, GDS, and DD. Interpretation followed CLSI M100-S34 breakpoints (MIC ≤ 0.25 mg/L or zone diameter ≥ 23 mm = susceptible). BMD served as the reference to calculate categorical agreement (CA), essential agreement (EA), major errors (ME), and very major errors (VME). Lefamulin exhibited potent activity against *S. aureus*, with 98.6% (416/422) of isolates classified as susceptible by BMD; six isolates (four MSSA and two MRSA) were non-susceptible. Compared with BMD, GDS showed 100% CA and 92.2% EA and yielded no VME and ME, and MICs were 0.49 log_₂_ lower than BMD. Disk diffusion with the 20 µg lefamulin disk achieved 99.5% CA (421/422), with a VME rate of 16.7% (1/6) and an ME rate of 0.2% (1/416). Lefamulin GDS fulfilled CLSI criteria but systematically underestimated MICs; BMD confirmation is advised for GDS MIC = 0.25 mg/L. Disk diffusion showed excellent agreement, yet its VME rate surpassed the CLSI threshold owing to scarce non-susceptible isolates; any non-susceptible result by disk should be verified by BMD.

**IMPORTANCE:**

Lefamulin is a new antibiotic active against methicillin-resistant *Staphylococcus aureus*, but simple susceptibility tests are lacking. We compared two easy-to-perform methods with reference broth microdilution for 422 *S. aureus* clinical isolates. Both showed high accuracy after on-site confirmation of borderline results and can be immediately implemented in routine laboratories to guide appropriate lefamulin therapy and help contain resistance.

## INTRODUCTION

*Staphylococcus aureus* (*S. aureus*) is a leading human pathogen that asymptomatically colonizes the skin and mucosal surfaces of 20%–30% of healthy individuals (1, 2). It can progress from colonization to invasive infections involving virtually every organ system, including skin and soft tissue, bone and joint, lung, bloodstream, endocardium, and indwelling medical devices (3–5). The global dissemination of methicillin-resistant *S. aureus* (MRSA) has been accompanied by escalating virulence and multidrug resistance, positioning this organism among the foremost causes of infection-related mortality and posing a formidable therapeutic challenge (6, 7). International surveillance programs consistently report MRSA isolation rates exceeding 20% (8, 9). In China, the 2024 CHINET Antimicrobial Resistance Surveillance Network (www.chinets.com) documented an MRSA prevalence of 32.3% among clinical isolates collected from 78 hospitals across China, including 72 tertiary-care and 6 secondary-care institutions (10). The presence of the mecA gene in MRSA encodes the low-affinity penicillin-binding protein PBP2a, conferring high-level resistance to all β-lactams and frequently to additional antimicrobial classes (11). Consequently, conventional therapeutic options have become increasingly restricted, underscoring the urgent need for novel agents that either exploit unprecedented mechanisms of action or demonstrate potent, targeted activity against MRSA.

Lefamulin is available for both intravenous infusion and oral tablets, allowing convenient step-down therapy (12). In the United States, it is Food and Drug Administration (FDA)-approved only for community-acquired bacterial pneumonia (CABP) with a 5- to 7-day course (13, 14). In China, the National Medical Products Administration granted marketing authorization in July 2025 (15), but nationwide susceptibility data remain scarce. Lefamulin is the first-in-class semi-synthetic pleuromutilin antibiotic that selectively binds to the A- and P-sites within the peptidyl-transferase center of bacterial 23S rRNA on the 50S ribosomal subunit, thereby preventing correct transfer RNA positioning and halting peptide-chain elongation (16). This bivalent interaction strengthens drug–ribosome affinity and markedly lowers the propensity for both cross-resistance and inducible resistance (17). *In vitro* studies demonstrate potent activity against gram-positive cocci and atypical pathogens, including multidrug-resistant MRSA, vancomycin-resistant enterococci (VRE), and penicillin-resistant *Streptococcus pneumoniae* (18). Global multicenter surveillance report MIC_₅₀/₉₀_ for *S. aureus* of 0.06/0.12 mg/L with 99.6% susceptible; among MRSA the corresponding values are 0.06/0.12 mg/L and 99.4% susceptible (19), while data for methicillin-susceptible *S. aureus* (MSSA) in China show 0.06/0.06 mg/L and 100% susceptibility (20). These findings have underpinned recommendations for lefamulin in CABP guidelines as an option for *S. aureus*, including MRSA, when indicated (21, 22).

Accurate and reproducible antimicrobial susceptibility testing (AST) is a prerequisite for the rational clinical use of lefamulin. At present, four methodologies are available: disk diffusion (DD), gradient diffusion strip (GDS), reference broth microdilution (BMD), and automated systems. Disk diffusion and GDS are inexpensive, technically undemanding, and already implemented in most Chinese clinical microbiology laboratories for single-patient testing. Although BMD is endorsed as the gold standard by both CLSI and EUCAST, its labor-intensive protocol and requirement for highly trained personnel limit routine adoption, whereas automated platforms—despite their high-throughput capability—rarely include lefamulin on their drug menus. Although VITEK 2 AST-Gram Positive Lefamulin (FDA 510(k) K234000, March 2023) now offers an automated option, disk diffusion and GDS remain the most rapidly deployable choices for laboratories without such high-throughput platforms (23). Notably, CLSI currently provides lefamulin breakpoints only for *S. aureus* (MIC ≤ 0.25 mg/L; zone ≥ 23 mm with 20 µg disks); no intermediate or resistant categories are defined (24). In contrast, EUCAST designates susceptible (S) ≤ 0.25 mg/L and resistant (R) > 0.25 mg/L and uses 5 µg disks (zone ≥ 23 mm = S, < 23 mm = R), likewise without an intermediate category (25). Large-scale, systematic evaluations of these AST modalities for lefamulin are still scarce. We therefore undertook the present study to assess the accuracy and reproducibility of GDS and disk diffusion relative to BMD in determining lefamulin susceptibility among 422 recent clinical isolates of *S. aureus*, thereby providing an evidence base for method selection and data interpretation in the routine laboratory.

## MATERIALS AND METHODS

### Bacterial isolates

A total of 422 non-duplicate *S. aureus* clinical isolates were consecutively collected from December 2023 to December 2024 at the Affiliated Tai’an Central Hospital of Qingdao University. Only the first isolate per patient per anatomical site was retained; 256 (60.7%) were MSSA, and 166 (39.3%) were MRSA. The specimens of origin were wound/abscess swabs (210/422, 49.8%), sputum (105/422, 24.9%), sterile body fluids (50/422, 11.8%), blood cultures (41/422, 9.7%), and other sources (16/422, 3.8%). Species identification was performed with matrix-assisted laser desorption/ionization time-of-flight mass spectrometry (Autof ms1000, Autobio, China). MRSA was defined as an oxacillin MIC ≥ 4 mg/L according to CLSI M100-34th edition (24). All isolates were verified, suspended in brain–heart infusion broth with 15% glycerol, and stored at −80°C until tested. Prior to each susceptibility assay, isolates were sub-cultured twice on sheep-blood agar to ensure purity and viability.

### Antimicrobial susceptibility testing

Lefamulin GDS and 20 µg disks (range 0.016–256 mg/L) were obtained from Liofilchem, Italy (FDA 510(k) K200308, cleared for S. aureus [MSSA only]; off-label use for MRSA under CLSI criteria). Pure lefamulin powder was purchased from Shanghai Zhenzhun Biotech. BMD, performed according to CLSI M07 (12th ed.) (26), served as the reference method. Twofold serial dilutions (0.015–32 mg/L) were prepared in cation-adjusted Mueller–Hinton broth (CAMHB) in 96-well microtiter plates (100 µL/well). The CAMHB was bought from the Becton, Dickinson Company (USA). A 0.5 McFarland suspension (approximately 1 × 10⁸ CFU/mL) was prepared and diluted 1:100 in CAMHB to approximately 1 × 10⁶ CFU/mL. Fifty microliters of this diluted inoculum was added to each 50 µL drug-containing well, yielding a final concentration of approximately 5 × 10^5^ CFU/mL. Plates were incubated at 35°C in ambient air for 16–20 h; the MIC was defined as the lowest concentration preventing visible growth.

Disk diffusion and GDS testing were performed on the same Mueller–Hinton agar plates (Antu Bio, China) in accordance with CLSI M02 (15th ed.) (27). Within 15 min of preparing the 0.5 McFarland suspension, a sterile cotton swab was dipped, excess fluid removed, and the entire MH plate surface streaked in three directions. A lefamulin GDS strip and a 20 µg disk were aseptically applied to the same plate, which was then incubated at 35 ± 2°C in ambient air for 16–20 h. The GDS MIC was read at the lowest concentration giving 100% inhibition of growth. Zone diameters were measured to the nearest millimeter with a digital caliper. All tests were performed in parallel from a single 0.5 McFarland suspension. Results were read independently by two investigators; concordant readings were accepted. When discrepancies occurred, a third reader evaluated the plates. If two of the three readings agreed, that value was recorded; if all three differed, the test was repeated. Interpretation followed the 2024 CLSI breakpoints (24): susceptible, MIC ≤ 0.25 mg/L or inhibition zone ≥ 23 mm. Quality control was performed with *S. aureus* ATCC 25923 (disk zone 26–32 mm) and ATCC 29213 (MIC 0.06–0.25 mg/L); values outside these ranges prompted repeat testing. For any isolate yielding discordant results between BMD, GDS, and DD, all three methods were repeated in parallel from the same 0.5 McFarland suspension; only reproducible discrepancies were recorded.

### Statistical analysis

BMD served as the comparator for evaluating GDS and disk diffusion. The following performance metrics were calculated: categorical agreement (CA): percentage of isolates for which the test method (GDS or disk) yielded the same susceptible/non-susceptible category as BMD, using CLSI 2024 breakpoints (≤0.25 mg/L = susceptible). Essential agreement (EA): percentage of isolates for which the GDS MIC was within ± one twofold dilution of the BMD MIC. Major error (ME): percentage of isolates classified as susceptible by BMD but non-susceptible by the test method (false-resistant). Very major error (VME): percentage of isolates classified as non-susceptible by BMD but susceptible by the test method (false-susceptible). Acceptable performance was predefined as EA ≥ 90%, CA ≥ 90%, ME ≤ 3%, and VME ≤ 1.5% (28). Following ISO 20776-2, (29) log_₂_-transformed MICs were used to calculate the difference (Δ) for each isolate: Δ = log_₂_(GDS MIC) − log_₂_(BMD MIC). Mean bias, standard deviation, and 95% confidence interval were computed from Δ; ±0.5 log_₂_ (approximately ±30%) bias is considered the commonly cited bias limit. The BMD-MIC distributions of MRSA and MSSA were compared using the Mann–Whitney *U* test (SPSS 26.0) with a two-tailed α of 0.05.

## RESULTS

### BMD reference results

Lefamulin MICs for the 422 *S*. *aureus* isolates ranged from ≤0.015 to >32 mg/L; MIC_₅₀_ and MIC_₉₀_ were 0.06 and 0.125 mg/L, respectively, with an overall susceptibility rate of 98.6% (416/422). MIC distributions were identical for MSSA (*n* = 256) and MRSA (*n* = 166): MIC_₅₀/₉₀_ 0.06/0.125 mg/L and susceptibility 98.4% (252/256) versus 98.8% (164/166) (Table 1). Mann–Whitney *U* test showed no statistically significant difference between the BMD–MIC distributions of MRSA and MSSA (*P* = 0.65).

**TABLE 1:** MIC distribution of lefamulin against *S. aureus* (BMD, mg/L)^*a*^

Organism	No. of isolates with lefamulin MIC (mg/L) of:														MIC_50_	MIC_90_
	≤0.015	0.03	0.06	0.125	0.25	0.5	1	2	4	8	16	32	>32	Total		
*S. aureus*	19	44	237	116	0	1	0	0	0	0	3	0	2	422	0.06	0.125
MSSA	12	26	147	67	0	1	0	0	0	0	1	0	2	256	0.06	0.125
MRSA	7	18	90	49	0	0	0	0	0	0	2	0	0	166	0.06	0.125

^*a*^ MIC, minimum inhibitory concentration; BMD, broth microdilution; MIC_50_, MIC for inhibiting 50% of the isolates; MIC_90_, MIC for inhibiting 90% of the isolates; MSSA, methicillin (oxacillin)-susceptible *S. aureus*; MRSA, methicillin-resistant *S. aureus*.

### Agreement between GDS and BMD for *S. aureus*

**Fig 1:**
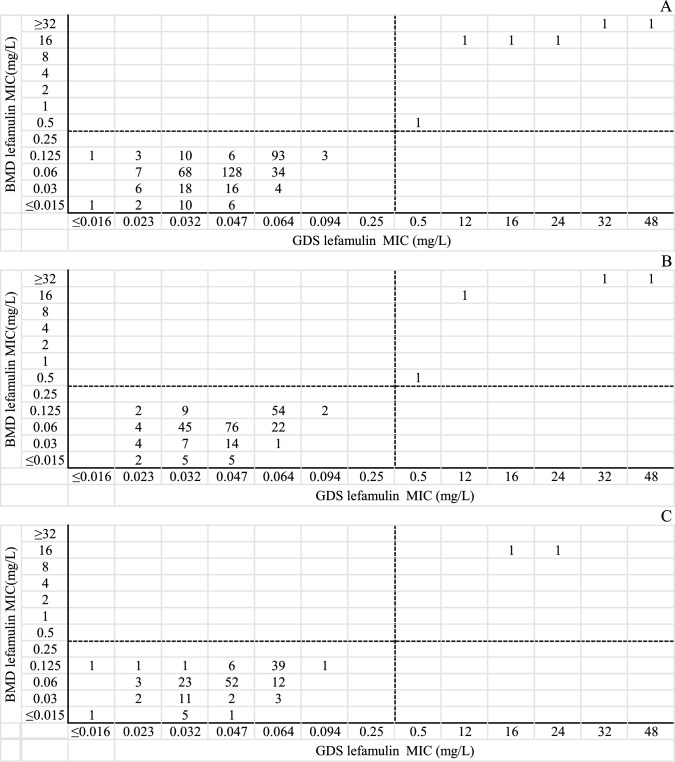
Scatter plot comparing the MIC values of lefamulin determined by GDS with the results of reference BMD method when testing *S. aureus*. Dotted lines indicate lefamulin breakpoints (CLSI). (**A**) All *S. aureus* (*n* = 422); (**B**) MSSA (*N* = 256); (**C**) MRSA (*N* = 166).

GDS MICs ranged from ≤0.016 to 48 mg/L (MIC_₅₀_, 0.047 mg/L; MIC_₉₀_, 0.064 mg/L), yielding a susceptibility rate of 98.6% (416/422). Compared with BMD, CA was 100%, and EA was 92.2% (389/422), with zero ME and zero VME. Subgroup analysis of MSSA and MRSA gave identical performance (Table 2, Fig. 1). All metrics fulfilled CLSI acceptability criteria (CA ≥ 90%, ME ≤ 3%, VME ≤ 1.5%); EA was also ≥90%. The bias assessment between GDS and reference BMD is summarized below. Bias analysis according to ISO 20776-2 showed that the GDS gradient strip underestimated MICs by a mean of −0.49 ± 0.68 log_₂_ dilutions (95% CI: −0.56 to −0.42). Although 92.2% (389/422) of isolates fell within ±1 log_₂_ dilution (EA ≥ 90%), the systematic negative shift approached the ±0.5 log_₂_ (approximately ±30%) bias limit considered acceptable for routine AST devices, warranting confirmation of isolates with MICs near the breakpoint.

**TABLE 2:** Concordance of different methods for detecting lefamulin susceptibility in *S. aureus^a^*

Organism	MTS					DD			
	Number	ME	VME	EA	CA	Number	ME	VME	CA
*S. aureus*	422	0	0	389 (92.2%)	422 (100.0%)	422	1 (0.2%)	1 (16.7%)	420 (99.5%)
MSSA	256	0	0	236 (92.2%)	256 (100.0%)	256	1 (0.4%)	1 (25.0%)	254 (99.2%)
MRSA	166	0	0	153 (92.2%)	166 (100.0%)	166	0	0	166 (100.0%)

^*a*^ ME, major error; VME, very major error; MTS, MIC Test Strip; DD, disk diffusion.

### Agreement between disk diffusion and BMD for *S. aureus*

**Fig 2:**
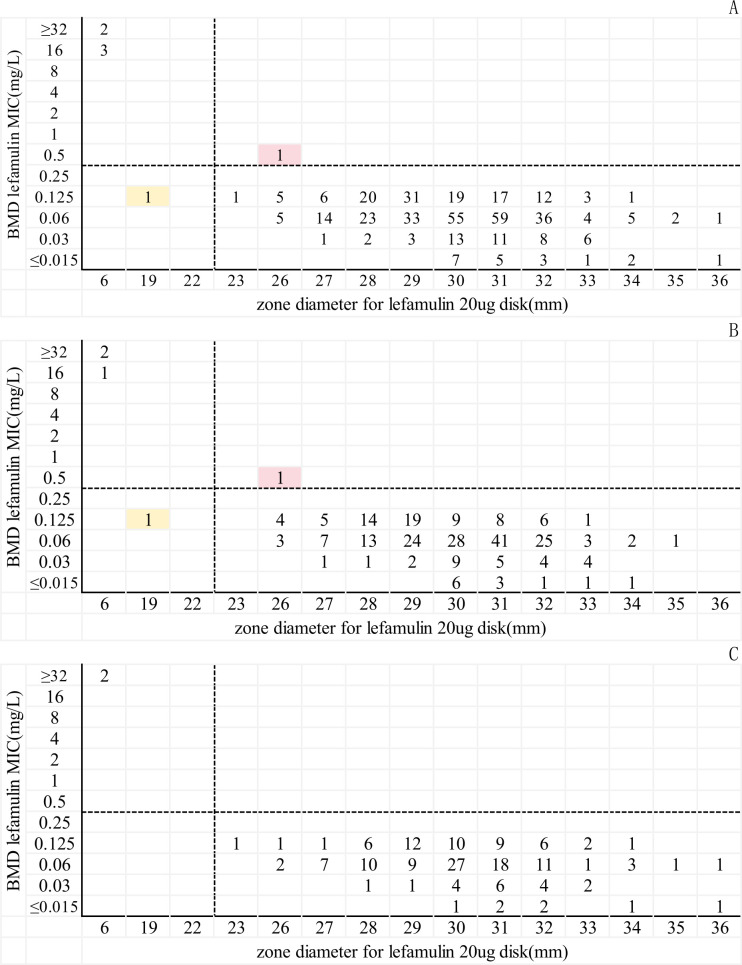
Scatter plot comparing the MIC values of lefamulin determined by the inhibition zone diameters of disk diffusion with the results of reference BMD when testing *S. aureus*. The orange background indicates that ME occurred for the DD compared with the BMD. The red background indicates that VME occurred for the DD compared with the BMD. Dotted lines indicate lefamulin breakpoints (CLSI). (**A**) All *S. aureus* (*n* = 422); (**B**) MSSA (*N* = 256); (**C**) MRSA (*N* = 166).

Inhibition zone diameters were 6–36 mm, giving a susceptibility rate of 98.6% (416/422). Overall, CA was 99.5% (420/422), ME 0.2% (1/416), and VME 16.7% (1/6). For MSSA, CA 99.2% (254/256), ME 0.4% (1/252), and VME 25.0% (1/4); for MRSA, CA 100% with zero ME and VME (Table 2, Fig. 2).

## DISCUSSION

The relentless spread of MRSA and other multidrug-resistant lineages has rendered many front-line agents obsolete, posing a direct threat to public health security (30). The emergence of vancomycin-intermediate or vancomycin-resistant strains, together with isolates non-susceptible to linezolid or daptomycin, has further eroded therapeutic options and jeopardized patient outcomes (31–33). Combating this crisis requires not only the accelerated discovery of novel antibiotics but also the continuous surveillance of resistance dynamics, thereby furnishing policy-makers and healthcare institutions with real-time, evidence-based data to guide targeted containment strategies (34). Lefamulin, with its potent *in vitro* activity against MRSA and a short treatment course (intravenous or oral), has been shown to reduce length of hospital stay, conserve medical resources, and alleviate healthcare expenditure, emerging as a valuable new weapon against antimicrobial resistance (35).

Using BMD as the benchmark, we systematically evaluated the reliability of GDS and disk diffusionDD for lefamulin against 422 *S*. *aureus* isolates. The MIC_₅₀/₉₀_ observed here (0.06/0.125 mg/L) mirrors those reported by the global SENTRY program (0.06/0.12 mg/L) (19) and the 2024 CHINET surveillance in China (0.06/0.125 mg/L) (36), confirming that contemporary local isolates remain highly susceptible to lefamulin. Importantly, the MIC distributions of MRSA and MSSA were statistically indistinguishable, indicating that *mecA*-mediated resistance does not compromise lefamulin activity—an observation consistent with the drug’s distinct ribosomal target (17). The present study confirmed a 98.6% susceptibility rate for lefamulin, consistent with global surveillance by Paukner et al. (19) and Wu et al. (20), further attesting to the high susceptibility of contemporary local isolates to this novel agent.

GDS integrates the principles of both dilution and diffusion techniques, offering simplicity of use while generating an MIC value—an advantage for processing individual clinical isolates. To our knowledge, this is the first performance evaluation comparing lefamulin GDS with BMD against a large collection of *S. aureus* clinical isolates. Our data demonstrate high concordance: categorical agreement 100%; essential agreement 92.2% (389/422), with zero major or very major errors—all within predefined acceptance limits. Although the GDS met CLSI categorical criteria, its systematic −0.49 log_₂_ negative bias could still produce VME at the breakpoint. Because lefamulin susceptibility is defined as ≤0.25 mg/L, any GDS result of 0.25 mg/L was retested by BMD, and the BMD value was used for the final report. These findings indicate that GDS can provide laboratories with a practical and readily implementable option to generate lefamulin MIC data without full automation, while maintaining clinical reliability.

Disk diffusion remains the most widely used AST format in Chinese clinical laboratories because it is inexpensive, rapid, and technically undemanding. In the present study, the test achieved 99.5% categorical agreement with BMD for *S. aureus*, mirroring the 99% reported by Cao et al. (37). We documented one ME (0.24%, 1/416) and one VME (16.7%, 1/6). The ME isolate exhibited a 19 mm zone (categorized as non-susceptible) but the MIC of 0.125 mg/L by BMD (susceptible); the VME isolate had a 26 mm zone (categorized as susceptible) yet an MIC of 0.5 mg/L by BMD (non-susceptible). Owing to the paucity of non-susceptible strains (*n* = 6), a single misclassification inflated the VME rate above the CLSI acceptable limit (3%). Thus, DD reliably identifies susceptible isolates and can be used for routine screening; however, any strain classified as non-susceptible or with a zone diameter ≤23 mm should be retested by BMD to avoid errors near the breakpoint. To reduce random error in routine practice, we recommend selective dual reading and concurrent QC with reference strains: any disk zone diameter ≤23 mm or GDS MIC = 0.25 mg/L is independently reviewed by a second technologist; if still ambiguous, the isolate is retested by BMD. Because commercial automated systems containing lefamulin are still scarce, manual disk diffusion and GDS remain the only practical options in the early post-marketing phase; they are inexpensive, easy to perform, and particularly valuable for lower-tier laboratories. Nevertheless, this study still has several limitations. All isolates originated from a single institution, and only one manufacturer’s disks and GDS strips were evaluated; product-specific performance could therefore bias the findings. Future work should compare reagents from multiple vendors and recruit isolates from multicenter surveillance networks to confirm the generalizability of lefamulin AST results.

In summary, using BMD as the reference, we systematically compared the reliability of GDS and disk diffusion for lefamulin against *S. aureus*. Lefamulin retained potent activity. GDS showed 100% CA and 92.2% EA with neither ME nor VME; only isolates with an MIC of 0.25 mg/L required confirmation by BMD, while all other results could be reported directly, supporting the routine use of GDS for quantitative testing. Disk diffusion achieved 99.5% CA with a 0.2% ME rate within CLSI limits; owing to the scarcity of non-susceptible isolates (*n* = 6), a single misclassification yielded a 16.7% VME rate. Thus, strains giving a zone diameter ≤ 23 mm or classified as non-susceptible should be retested by BMD. Larger, multicenter studies comparing products from multiple vendors are warranted to confirm the generalizability and robustness of these assays.

## Data Availability

All data generated or analyzed during this study are included in this published article.
